# Editorial: Secondary analysis of national nutrition surveys: from science to policy

**DOI:** 10.3389/fnut.2023.1297915

**Published:** 2023-10-06

**Authors:** Julie M. Hess, Yong Zhu

**Affiliations:** ^1^USDA-ARS Grand Forks Human Nutrition Research Center, Grand Forks, ND, United States; ^2^Wayzek Science, St.Paul, MN, United States

**Keywords:** nutrition, policy, surveys, epidemiology, food-based dietary guidelines, dietary guidance

## 1. Introduction

National nutrition survey data provide insight into nutrient intake, food intake, and health status at a population level. Many countries, including the United States, South Korea, Mexico, Japan, and several European countries (1) conduct national nutrition surveys, which generate important context for targeted nutrition policies including food-based dietary guidance (FBDGs). In the US, data from the National Health and Nutrition Examination Survey (NHANES) are used as part of the evidence base for updating public health policies, including the Dietary Guidelines for Americans (DGA). NHANES data inform recommended healthy dietary patterns in the DGA, help identify nutrients of concern for under- and over-consumption across the population, and provide early signals of nutritional insufficiencies among specific age, sex, and/or socioeconomic groups. These publicly available data function as a cost-effective resource for vital nutrition information.

The goal of this Research Topic is to share the latest findings from secondary analyses of national nutrition surveys across the globe with a special focus on how research findings may be translated to support the development of nutrition and public health policies.

The eight articles in this Research Topic come from diverse geographic areas and showcase the nutrition challenges faced by and different policies enacted in different regions of the world. Studies from Ethiopia and India focus on new data and analytical methods to evaluate nutrient insufficiencies. Studies from Iran focus on healthy dietary patterns to reduce chronic disease incidence, and a study of European countries evaluates the potential of front-of-pack labeling schemes to promote improved dietary choices. Two US-based studies evaluate the impact of consuming specific foods on nutrient intake and health markers, while a third study from the US assesses the ability of vegetarian patterns to provide nutritionally balanced diets. Brief summaries of these studies are presented by geographic area below.

## 2. Ethiopia

Roba and Başdaş article examines the trends and predictors of wasting and stunting in children using data from the Ethiopian Demographic and Health Survey. The study revealed a significant decline in prevalence of both wasting and stunting among Ethiopian children from 2000 to 2019 and identified several ongoing risk factors, also highlighting opportunities for implementation of intervention programs to eradicate these conditions. Data for this study came from Ethiopia's Demographic and Health Surveillance (DHS) program. The DHS report was also used to provide context for rates of undernutrition and malnutrition in Ethiopia for their first national nutrition guidelines released in 2022 (2).

## 3. European countries

A study from Mertens and Peñalvo evaluates the potential of Nutri-Score, a front-of-pack nutrient “grading” system, to improve dietary intakes across European countries among adolescents, adults, and older adults. Nutri-Score remains a voluntary addition to front-of-pack labels in several countries, however, as Mertens and Peñalvo discuss, the European Commission “intends to propose a harmonized mandatory front-of-pack label at the European Union level.” This article evaluates the current intake of food items by Nutri-Score grade by age range, sex, and country and reveals significant differences in consumption of energy from foods with Nutri-Score grades of A, B, C, D, and E across European countries. Estonia had the greatest energy intake from A-classified foods, and Latvia had the highest energy intake from E-classified foods. Older adults and females also tended to consume more energy from foods with “better” Nutri-Score grades (A-class foods). More research is needed to determine the potential of Nutri-Score labeling to improve the percentage of energy intake coming from nutrient-dense foods across all sectors of the European population.

## 4. India

Ghosh et al. study focused on nutrients of concern for the Indian population using biomarker data from the comprehensive national nutrition survey (CNNS) to assess the risk of deficiencies of retinol, zinc, folate, and vitamin B12 in children. Instead of relying on pre-determined cut-off values for insufficiencies or deficiencies, which are usually derived from populations in high-income countries, Ghosh et al. employed a risk-based probability approach that can define locally relevant cut-off values for nutrient deficiency and, therefore, may be more suited for low- and middle-income countries. This method could provide important background information for future iterations of India's dietary guidance, last updated in 2011 (3).

## 5. Iran

Beigrezaei et al. analyzed data from two Iranian cohort studies to compare three statistical methods in identifying dietary patterns associated with risk of hypertension. The study revealed that reduced rank regression best identified dietary patterns associated with hypertension incidence. Nouri et al. examined the associations among intake of fruits, vegetables, and dairy products with body weight status using data from a national Stepwise Approach to Surveillance Survey. The benefits of consuming fruits, vegetables, and dairy products emphasized in Nouri et al. conclusion echoes the focus of Iran's dietary guidance, which encourages consuming raw and cooked vegetables at every meal, three daily servings of fruit, and at least one daily serving of dairy foods (4).

## 6. United States

Zhu et al. examined associations among cereal consumption, meal cost, and dietary outcomes using NHANES data. Breakfast skipping did not result in meal cost savings (per calorie) but was associated with decreased intake of critical nutrients and food groups. Low-cost nutrient-dense food options remain an important option in federal nutrition assistance programs. Pang et al. also used NHANES data to investigate associations between coffee consumption and frailty in older adults. This study found a non-linear relationship showing a reduction of frailty risk with lower caffeinated coffee consumption, which may be a useful consideration for dietary guidance targeted to older adults. Finally, Hess et al. food pattern modeling study indicates that nutritionally adequate pescatarian, lacto-vegeterian, and pescavegan diets can be developed with modifications to the Healthy Vegetarian Dietary Pattern from the 2020 DGA. The results of this study indicate the flexibility of recommended patterns in the DGA to accommodate dietary preferences.

## 7. Conclusions

These studies highlight multiple opportunities for future research and the continuous need to update FBDGs to reflect the latest evidence base. These remain important aims as we near the end of the United Nations Decade of Action on Nutrition (2016–2025) and identify new goals to support global public health in the years to come (5).

## Author contributions

JH: Writing—original draft, Writing—review and editing. YZ: Writing—original draft, Writing—review and editing.
